# Primary mucinous cystadenocarcinoma of the renal pelvis misdiagnosed as ureteropelvic junction stenosis with renal pelvis stone: a case report and literature review

**DOI:** 10.1186/s12957-015-0739-7

**Published:** 2015-11-26

**Authors:** Dong Seok Han, Seung Mo Yuk, Chang Shik Youn, Geon Park, Hae Joung Sul, Hoon Jang

**Affiliations:** Department of Urology, College of Medicine, The Catholic University of Korea, Seoul, Republic of Korea; Department of Radiology, College of Medicine, Catholic University of Korea, Seoul, Republic of Korea; Department of Pathology, College of Medicine, Catholic University of Korea, Seoul, Republic of Korea; Department of Urology, Daejeon St. Mary’s Hospital, College of Medicine, The Catholic University of Korea 64, Daeheung-ro, Jung-gu, Daejeon, 34643 Republic of Korea

**Keywords:** Kidney, Mucinous adenocarcinoma, Renal pelvis

## Abstract

**Background:**

Primary mucinous adenocarcinoma of the renal pelvis is extremely rare, with only ~100 cases reported till now. Its presumed pathogenesis includes glandular metaplasia of the urothelium of the calyces and the pelvis and malignant transformation of the metaplasia. Unfortunately, it has no characteristic symptoms or radiological features. We report a case of primary mucinous adenocarcinoma of the renal pelvis misdiagnosed as ureteropelvic junction stenosis with a renal pelvis stone.

**Case presentation:**

A 50-year-old man presented with discomfort in his right flank after a fall. A physical examination was normal except mild costovertebral angle tenderness on the right side. The results of most laboratory tests were within normal limits. Plain radiography of the kidneys, ureter, and urinary bladder showed a large radio-opaque mass in the right kidney. Abdominal computed tomography showed a hyperdense mass with 2.62 × 5.70 cm size in the right renal pelvis and severe hydronephrosis and cortical thinning. Diuretic-enhanced 99mTc DTPA renal scanning showed that the relative function of the right versus the left kidney was 20 versus 80 %. On the basis of the imaging findings, kidney dysfunction due to ureteropelvic junction stenosis with a large stone was initially diagnosed.

However, the drained urine volume was almost zero, and gelatinous material was aspirated when percutaneous nephrostomy was performed for decompression of hydronephrosis. Although the cytopathology of gelatinous material was negative for malignancy, we could not rule out other disease, such as hidden malignancies of the kidney.

We therefore performed radical nephrectomy, and pathological examination of the kidney uncovered a mucinous cystadenocarcinoma in the renal pelvis. A bone scan and positron emission tomography showed no evidence of other malignancies, metastasis, or remnant cancer. The patient has been well, without evidence of tumour recurrence or metastasis, for 20 months after surgery.

**Conclusions:**

Primary mucinous adenocarcinomas of the renal pelvis are extremely rare, and most are diagnosed via post-operative analysis of resected specimens. Although preoperative diagnosis is difficult, urologists should consider the possibility of primary mucinous adenocarcinoma in patients with severe hydronephrosis accompanied by renal stones and chronic inflammation

## Background

Malignant tumours arising from the renal pelvis include transitional cell carcinoma (85–90 %), squamous cell carcinoma (10–15 %), and adenocarcinoma (<1 %). Adenocarcinomas of the renal pelvis are rare and are classified as tubulovillous, mucinous, or papillary non-intestinal [1, 2]. Primary mucinous adenocarcinoma of the renal pelvis, first described in 1960 by Hasebe et al. [3], is especially rare. Till date, only ~100 cases have been reported [4], most from Asian countries [4–8]. Although its etiopathogenesis is unclear, it is thought to originate from intestinal metaplasia in the transitional epithelium [1, 5, 9, 10]. Unfortunately, it is difficult to diagnose preoperatively because there are no characteristic symptoms or laboratory and radiological findings. Herein, we report a rare case of primary mucinous cystadenocarcinoma of the renal pelvis initially misdiagnosed as ureteropelvic junction (UPJ) stenosis with a renal pelvis stone.

## Case presentation

### Patient and treatment

**Fig. 1:**
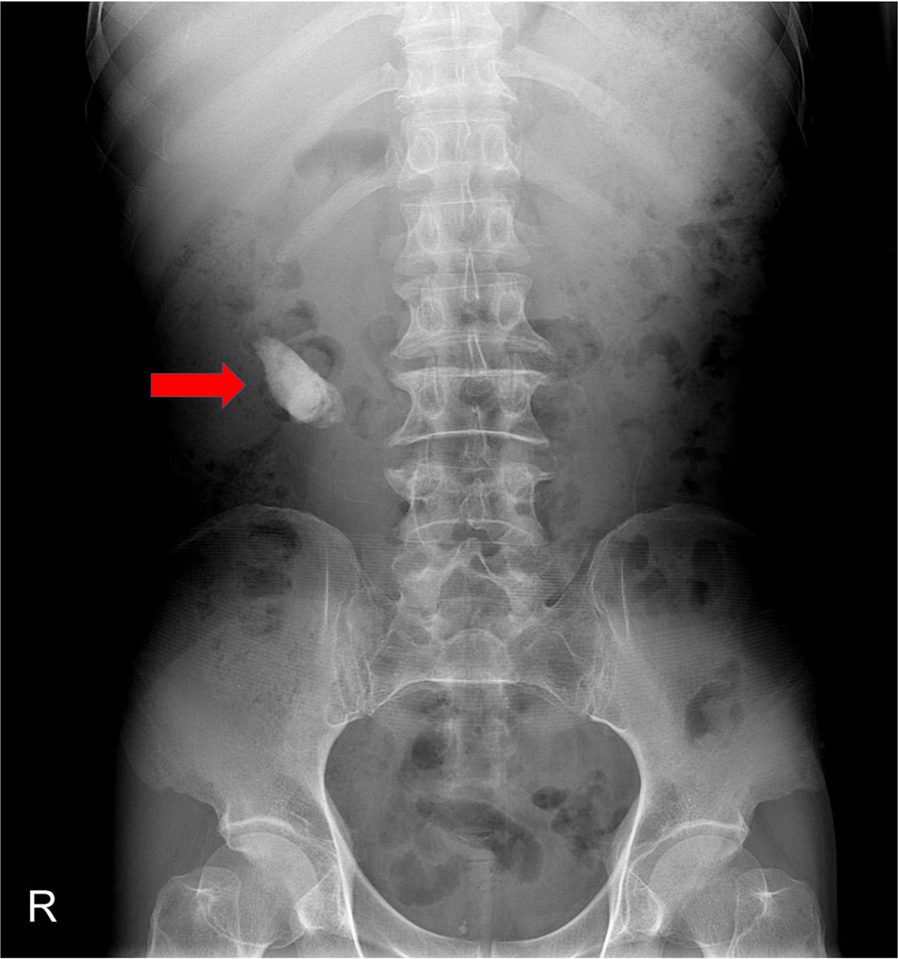
Plain radiography. Plain radiography of the kidney, ureters, and bladder (KUB) shows a radio-opaque mass in the right kidney (*red arrow*)

In September 2013, a 50-year-old man from Vietnam with an unremarkable medical history presented at our outpatient clinic with pain in his right flank after a fall. He also complained of voiding difficulty and nocturia. He had smoked one pack of cigarettes (20/pack) per day for 30 years, but did not have any respiratory symptoms. The physical examination was normal except for mild costovertebral angle tenderness on the right side. In the initial laboratory tests, there were no red or white blood cells in urinalysis and the results of the biochemistry tests were within normal limits. Chest radiography showed an old tuberculosis scar, and plain radiography of the kidneys, ureter, and urinary bladder showed a large radio-opaque mass in the right kidney (Fig. 1). Abdominal computed tomography (CT) showed a hyperdense mass of 2.62 × 5.70 cm in the right renal pelvis with severe hydronephrosis and cortical thinning. Diuretic-enhanced 99mTc DTPA renal scanning showed that the relative function of the right versus the left kidney was 20 versus 80 %. On the basis of imaging findings, kidney dysfunction due to ureteropelvic junction (UPJ) stenosis with a large renal pelvis stone was initially diagnosed.

We created a percutaneous nephrostomy (PCN) to decompress the hydronephrosis. Interestingly, the drained urine volume was almost zero and gelatinous material was aspirated via PCN catheter.

**Fig. 2:**
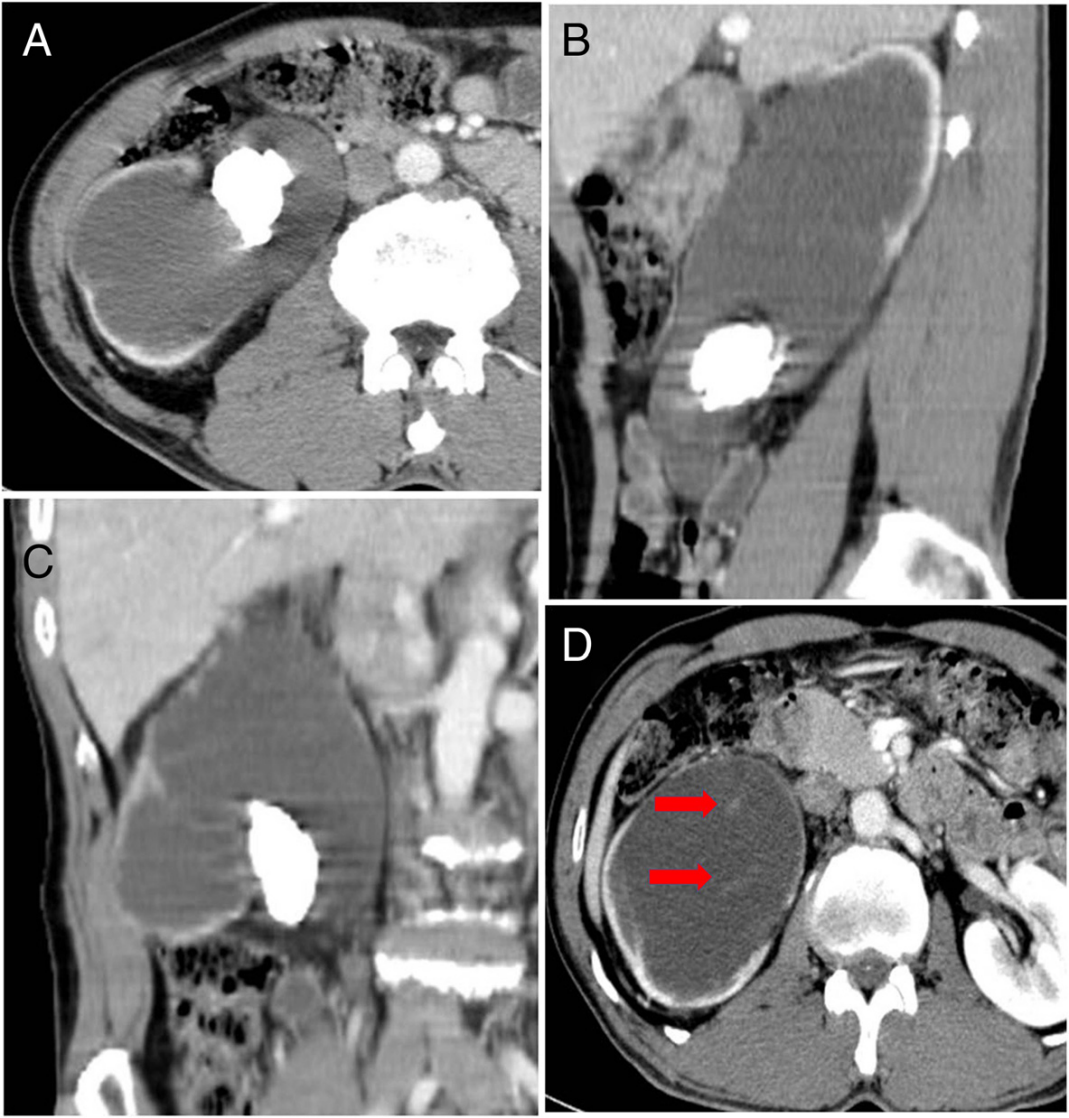
Abdominal computed tomography (CT). **a–c** Post-enhancement CT shows a hyperdense mass of 2.62 × 5.70 cm in the right renal pelvis. This mass did not obstruct UPJ directly, and the ureter below the stone was dilated. **a** Transverse view. **b** Sagittal view. **c** Coronal view. **d** Another section of the transverse view of the CT shows dense lines (*red arrows*) in the dilated right renal pelvis, suggesting septa

Urinary cytopathology of the gelatinous material indicated no malignancy. We reviewed the preoperative radiological images and noted that the stone did not obstruct the UPJ directly, although the stone was located in the renal pelvis. The upper ureter, located below the stone, was dilated (Fig. 2a–c), and a transverse view of the abdominal CT showed dense lines and unclear enhancement in the dilated right renal pelvis, suggesting septa (Fig. 2d). Although the urinary cytopathology was negative for malignancy, we could not exclude the possibility of other disease or hidden malignancies of the kidney. We performed a radical nephrectomy with a grossly safe resection margin of the ureter without an intraoperative frozen section study in the resected kidney and resected the margin of the ureter.

Pathological examination of the kidney after surgery revealed a mucinous cystadenocarcinoma in the renal pelvis. Endoscopy showed no other tumours in the gastroduodenal tract, and a bone scan and positron emission tomography-computed tomography (PET-CT) showed no evidence of other malignancies, metastasis, or remnant cancer.

The patient did not receive any post-operative radiation therapy or chemotherapy. He was followed up via urinalysis, biochemistry, urinary cytopathology, cystoscopic examination, and abdominal CT scan at 3-month intervals postoperatively for the first year and every 6 months thereafter and a yearly PET-CT. He has been well, without evidence of tumour recurrence or metastasis, for 20 months post-surgery.

### Pathology

Grossly, the kidney showed a cystically dilated pelvi-calyceal system with thinning of the parenchyma and contained mucoid material. At cross section, there was no definite mass-like lesion, and the cut surface had a soft consistency with a gelatinous appearance. The large stone was noted in the dilated lower calyx adjacent to the UPJ and did not obstruct the UPJ directly. The stone seemed to float in a mucin pool.

**Fig. 3:**
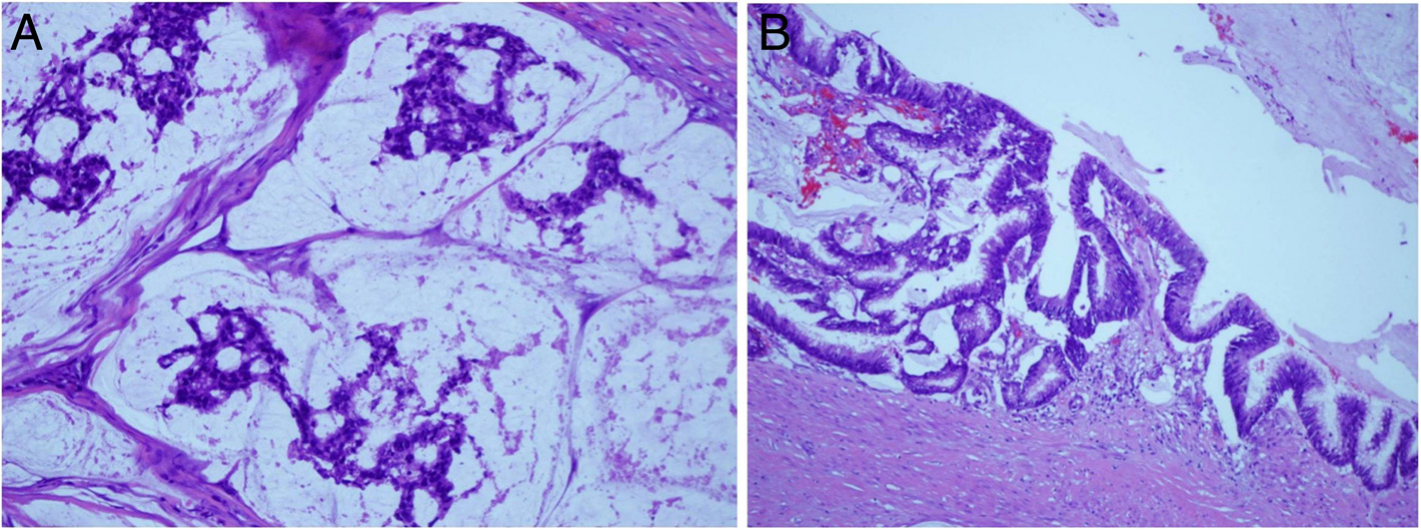
Haematoxylin and eosin staining. **a** Mucus-secreting cells are arranged in chains and surrounded by abundant extracellular mucus (×400). **b** The cystically dilated lesion is lined with multi-layered tall columnar mucinous epithelium (×200)

On light microscopic examination, the renal pelvis was composed of pools of mucus with clumps or strands of neoplastic glandular epithelium (Fig. 3a), and the pelvic mucosa consisted of tall columnar cells that tended to stratify into two or more layers, with perfuse irregular infolding and protrusions into the surrounding stroma (Fig. 3b). The tumour cells extended into the peripelvic fat tissue and were not found in the resection margin of the ureter.

### Discussion

The pathogenesis of primary mucinous adenocarcinoma in the renal pelvis is unclear. A possible mechanism involves glandular metaplasia of the urothelium that develops in response to injury (e.g. chronic irritation, inflammation, infection, hydronephrosis, or urinary calculi) and progresses to dysplasia and adenocarcinoma [1, 5, 7, 10]. In our case, we believed that the renal pelvis stone may have caused chronic inflammation in the renal pelvis, which then spread to the entire pelvis and ureter. This also induced stenosis of the ureter, resulting in hydronephrosis of the right kidney. Over time, this condition might have given rise to glandular metaplasia of the urothelium, leading to development of this tumour.

In previous reports, no characteristic symptoms of primary mucinous adenocarcinoma were noted. Most patients simply reported flank discomfort. A palpable abdominal mass may be a sign of late-stage disease [2, 11, 12]. In our patient too, there was no specific symptom except pain over the right flank and mild costovertebral angle tenderness on the right side on physical examination. We believe that it is impossible to suspect or diagnose this tumour through history taking and physical examination preoperatively.

Radiological studies including abdominal ultrasonography, intravenous pyelography, and abdominal CT may not be able to identify a malignant tumour [7]. According to Sisoda et al. [12] and Abbas et al. [2], no radiological features define a primary mucinous adenocarcinoma. In most reported cases, a non-function kidney, hydronephrosis, and renal calculi were noted in abdominal CT scans; these features are consistent with, but not conclusive of, malignancy. Moreover, most primary mucinous adenocarcinomas of the renal pelvis are diagnosed only after pathologic analysis of the resected specimen. Similarly, in our case, severe hydronephrosis with cortical thinning and a large renal pelvis stone were observed in abdominal CT scans, and the decreased kidney function was identified with diuretic-enhanced 99mTc DTPA renal scanning, which led to the initial diagnosis. However, in the review of the preoperative radiological images after indentifying gelatinous material aspiration without urine drainage via PCN catheter, the stone did not obstruct the UPJ directly, and the upper ureter, located below the stone, was dilated. Generally, in kidney dysfunction due to renal pelvis stone, the UPJ is directly obstructed by the stone. Therefore, we hypothesize that hydronephrosis without direct obstruction of the renal pelvis by a stone and ureter dilatation below the level of the stone without definite obstruction causes could be signs of this tumour.

In view of the presence of cysts containing large pools of mucin and gelatinous areas in most documented cases of mucin-secreting adenocarcinoma, Raphael et al. [11] and Abbas et al. [2] suggest that diagnosing carcinoma in this circumstance requires a strong clinical suspicion and that an intraoperative frozen section study or cytology may help confirm the diagnosis and planning the appropriate surgery. Unfortunately, cytopathology of the gelatinous material via preoperative PCN did not help to diagnose this tumour preoperatively in our case. However, we suggest that the preoperative Tru-Cut biopsy may help to detect this tumour and to decide the range of surgical treatment.

The recommended treatment for tumours in the renal pelvis is radical nephrectomy and total ureterectomy, including the intravesical area [13]. In our case, without an intraoperative frozen section study in the resected kidney and resection margin of the ureter, we performed a radical nephrectomy with a grossly safe resection margin of the ureter.

Although fortunately there was no tumour in the resection margin of the ureter, we believe that surgical treatment based on reasons of absence of malignancy in the preoperative cytopathology and decreased kidney function was an error in a patient who could have had other disease or hidden malignancies. We should have performed an intraoperative frozen section study of the kidney, along with a total ureterectomy including the intravesical area. We also suggest that the radical nephrectomy with total ureterectomy is necessary in patients who are suspected or diagnosed with adenocarcinoma of the renal pelvis or ureter.

Despite reports of good prognosis without recurrence even 3 or more years after surgery [5], the overall prognosis of patients with primary mucinous adenocarcinoma is poor, with ~50 % of the patients dying within 2 years of surgery [11, 12]. Local recurrence due to both spillage of tumour cells during surgical manipulations and downward seeding in the distal ureter has been reported [2, 11]. Fortunately, in our case, the patient has been well, without evidence of tumour recurrence or metastasis, for 20 months after surgery.

The guidelines regarding follow-up and surveillance are not yet established in primary mucinous adenocarcinoma of the renal pelvis and ureter. However, we suggest that the European Association of Urology (EAU) guidelines on urothelial carcinomas of the upper urinary tract [14] may help to evaluate the recurrence or metastasis of this tumour.

## Conclusions

Primary mucinous adenocarcinomas of the renal pelvis are extremely rare, and most are diagnosed via post-operative analysis of resected specimens. Although diagnosis of this tumour type is difficult preoperatively, urologists, pathologists, and radiologists should look carefully for any neoplastic growth in patients with a longstanding calculus. The possibility of primary mucinous adenocarcinoma in patients with severe hydronephrosis accompanied by renal stones and chronic inflammation should be kept in mind. Further investigation of the etiopathogenesis of this disease is required, as well as development of new diagnostic tools and effective treatment protocols.

## Consent

Written informed consent was obtained from the patient for publication of this case report and any accompanying images. A copy of the written consent is available for review by the Series Editor of this journal.

## Abbreviations

CT: computed tomography
PCN: percutaneous nephrostomy
PET-CT: positron emission tomography-computed tomography
UPJ: ureteropelvic junction
